# Kinetics of Cytotoxic Lymphocytes Reconstitution after Induction Chemotherapy in Elderly AML Patients Reveals Progressive Recovery of Normal Phenotypic and Functional Features in NK Cells

**DOI:** 10.3389/fimmu.2017.00064

**Published:** 2017-02-02

**Authors:** Jérôme Rey, Cyril Fauriat, Eloïse Kochbati, Florence Orlanducci, Aude Charbonnier, Evelyne D’Incan, Pascale Andre, François Romagne, Bernadette Barbarat, Norbert Vey, Daniel Olive

**Affiliations:** ^1^Département d’Hématologie, Institut Paoli-Calmettes, Marseille, France; ^2^Plateforme d’Immunomonitoring en Cancérologie de Marseille, Institut Paoli-Calmettes, Marseille, France; ^3^Centre de Recherche en Cancérologie de Marseille, INSERM U1068, Institut Paoli-Calmettes, Aix-Marseille Université, UM105, CNRS, UMR7258, Marseille, France; ^4^Innate-Pharma, Marseille, France; ^5^Mi-mAbs, Aix-Marseille Université, Marseille, France

**Keywords:** NK cells, acute myeloid leukemia, chemotherapy, activating receptors, NK functions

## Abstract

NK cells are defective in acute myeloid leukemia (AML) at diagnosis. Here, we studied the kinetic of expression of the major activating and inhibitory receptors of NK, CD8 T, and γδ T cells in patients undergoing chemotherapy (CT) for the treatment of AML (*n* = 29). We showed that NK cells are the main affected population at diagnosis and that expression of activating receptors is partially restored within a few weeks after CT. CD8 T cells and γδ T cells are only weakly affected at diagnosis. Killer cell immunoglobulin-like receptor expression by NK cells, but not NKG2A and CD85j, was downregulated. Interestingly, the development of NK cells appeared altered as the most immature CD56^bright^ NK cells were seriously underrepresented. Finally, we showed that NK cell functions were only partially restored 6 weeks after CT as degranulation capabilities of NK cells recovered, whereas cytokine production remained low. Our data point out NK cells as antitumor effectors peculiarly hampered by leukemic cells. This study may indicate a timeline when NK-mediated therapies or other immunotherapies could be performed, particularly for patients excluded of hematopoietic stem cell transplantation.

## Introduction

Immunity against cancer and in particular hematological malignancies relies on the capacity of effector immune cells to recognize and kill tumor cells and to alert other immune cells. The armed branch of the immune system responsible for tumor clearance encompasses NK cells, CD8^+^ αβ T cells (later referred to as CD8 T cells) and γδ T cells.

Activation of CD8^+^ αβ T cells requires the peptidic antigen-specific triggering of the TCR, in association with costimulatory molecules of the B7 family. γδ T cells recognize phospho-antigens originating from the metabolism/mevalonate pathway of cells, which is often up-regulated in cancer cells. Activation of mature CD8 T cells and γδ T cells can be improved by several activating coreceptors such as NKG2C, NKG2D, 2B4, and DNAM-1.

NK cells belong to the innate immune system and secrete many cytokines, such as GM-CSF, IL-10, or pro-inflammatory cytokines (IFN-γ and TNF-α), and chemokines. Activation of NK cells usually requires triggering of several cooperative receptors (1, 2). Some of them are specific for NK cells (natural cytotoxicity receptors, NKp30, NKp46, and NKp44), the others can be shared with cytotoxic T cells (3). Of note, NKG2C is the activating counterpart of NKG2A, and both recognize HLA-E. Activation of NK cells is balanced by inhibitory receptors, most of them recognizing classical HLA class I, or the non-classical class I HLA-E. Killer cell immunoglobulin-like receptors (KIRs) (CD158) are specific for class I HLA-A, B, and C, whereas NKG2A, a C-type lectin, recognizes HLA-E, and CD85j (LILRB1/ILT2) has a broad reactivity of HLA-I. Early immature NK cells express high levels of CD56 (CD56^bright^ NK cells), high levels of NKG2A, and do not express CD16. After an intermediate stage characterized by a dim expression of CD16, NK cells lose partially CD56 expression and start to express inhibitory or activating KIRs, while gradually losing NKG2A expression (CD56^dim^ NK cells) (4). These cells express high levels of CD16. Further maturation involves the acquisition of more KIRs, loss of NKG2A, and finally acquisition of CD57 (5, 6).

Reduced NK cell functions have been shown to promote cancer (7). NK cell importance in leukemia control has been evidenced by the pioneering study of Ruggeri and was followed since by many centers (8, 9). Hence, NK cells are found profoundly altered in solid cancers, such as breast cancer, neuroblastoma, and GIST, and in hematological malignancies such as multiple myeloma or acute myeloid leukemia (AML) (10–13). These phenotypic and functional alterations strongly suggest the need for tumor cells to hamper NK cell-mediated recognition (14). Although the mechanisms behind the interaction and destruction of leukemic cells are not yet clearly defined, the high rate of relapse in AML suggests these mechanisms are altered and profit to leukemic cells that can escape from the immune system (15, 16).

The current mainstay of AML treatment is based on conventional chemotherapy (CT) using anthracyclines and cytarabine, and hematopoietic stem cell transplantation (HSCT) for patients with poor prognosis features. With this treatment, about 50–80% of patients attain complete remission (CR). However, the 2-year survival is only 15–50% due to frequent relapses (17). Although a lot of them have poor-risk disease, HSCT is often not feasible because of comorbidities. There is thus an urgent need to develop new immunotherapeutic approaches that represent alternatives to HSCT such as vaccines, monoclonal antibodies, or immunomodulatory drugs (IMIDs). However, these approaches often require a functional immune system to facilitate the clearance of tumor cells. Therefore, it is relevant to monitor the status of immune cells, since so far, little is known about the effects of CT drugs on immune cells, with very few *in vitro* studies performed on cytotoxic T cells or NK cells (18, 19).

In this study, we analyzed the phenotype of peripheral blood NK cells, γδ T cells, and CD8 αβ T cells (CD8 T cells) of elderly patients treated with CT for AML. Blood samples were collected at diagnosis, remission, and various time points following consolidation CT in order to evaluate potential alterations following CT. Our data revealed important phenotypic alterations of NK cells, contrasting with limited phenotype alteration of γδ T cells and CD8 T cells. The most immature NK cell population was absent at diagnosis and recovered slowly after CT. NK cells presented low cytolytic activity at diagnosis that recovered with time, but their capacity to produce pro-inflammatory cytokines was durably impaired. Overall, these data provide the basic knowledge required for the design of clinical trials of immunotherapies for the treatment of AML in the elderly.

## Patients and Methods

### Patients

We enrolled 29 elderly patients (60–80 years old) with non-promyelocytic AML according to WHO criteria in first CR following induction CT (3 + 7 regimen). All patients have received an induction and one consolidation CT before inclusion. All patients received informed consent. The study was approved by a local ethics committee and the national institution [AFSSAPS (Agence Française de Sécurité Sanitaire des Produits de Santé), No DGS 2006/0396]. Patient peripheral NK, γδ T, and CD8 T cells were analyzed at diagnosis, the day before the second consolidation CT (W0), and every other week after treatment for 8 weeks (Figure S1 in Supplementary Material). Patient characteristics are presented in Table 1. All patients were in CR at W0. Induction CT was as follows: daunorubicin 45 mg/m^2^ D1–D3, cytarabine 100 mg/m^2^ D1–D7; consolidation CT 1 is as follows: daunorubicin 45 mg/m^2^ D1–D2, cytarabine 50 mg/m^2^ subcutaneous twice daily D1–D5; consolidation CT 2 is as follows: idarubicin 8 mg/m^2^ D1, cytarabine 50 mg/m^2^ subcutaneous BID D1–D5.

**Table 1:** **Characteristics of patients**.

Characteristic	*N*
Number of patients	29
**Age, years**	
Mean (SD)	70.17 (1.45)
Median [min–max]	70.00 [38.00–81.00]
≤65	4 (13.79)
>65	25 (86.21)
**Sex, *n* (%)**	
Male	19 (65.52)
Female	10 (34.48)
**FAB category, *n* (%)**	
M1	4 (13.79)
M2	8 (27.59)
M4	9 (31.03)
M5	4 (13.79)
M6	2 (6.90)
Unclassified	2 (6.90)
**Cytogenetics, *n* (%)**	
Normal	21 (72.41)
Favorable	1 (3.45)
Complex	7 (24.14)

Fifteen healthy donors (HD), age-matched, were used as controls and were obtained from the Etablissement Français du Sang. Median age of HD was 72.2 years [65.6–76.4] and the ratio F/M was 8/7. No major past clinical history was noticed for these donors.

### Phenotypic Studies

Peripheral blood samples from HD and AML patients were processed and cryopreserved until use. After thawing, PBMCs were processed for flow cytometry experiments. The antibodies used for these experiments are listed in Table S1 in Supplementary Material. 7-AAD was used as a live/dead discrimination marker. Protocols and FACS analysis were performed according to published protocols (1).

### Proliferation Assays

PBMCs were thawed up, washed twice in PBS, and incubated 20 min with 2.5 µM CellTrace Violet at 37°C. Cells were then washed twice in PBS before resuspension in RPMI containing 10% FCS, 100 UI/mL IL-2, and 10 ng/mL IL-15. After 6 days of culture, cells were harvested and prepared for flow cytometry analysis. The antibodies used for these experiments are listed in Table S1 in Supplementary Material.

### Degranulation and Cytokine Production Assays

PBMCs were thawed up and incubated overnight at 37°C with RPMI 10% FCS (complete medium) alone or with complete medium containing IL-2 (100 UI/mL) + IL-15 (10 ng/mL) or IL-12 (5 ng/ml) + IL-18 (10 ng/mL). Cells were then incubated with K562 cells (ratio 1:10) at 37°C for 4 h in the presence of GolgiPlug (Life Technologies). The antibodies used for these experiments are listed in Table S1 in Supplementary Material. Functional tests with NK cells at diagnosis could not be performed because of lack of material and because of the extremely low frequency of NK cells counts at this time point.

### Cytotoxicity Assays

NK cells were isolated using magnetic isolation kit (StemCell Technologies). The purity of NK cells was determined by flow cytometry and was >98%. K562 target cells were labeled with ^51^Cr (Perkin-Elmer). After three washes, NK cell cytotoxicity against the HLA class I-deficient K562 cell line was evaluated with a standard 4-h ^51^Cr-release assay at various effector/target ratios (10:1 and 2:1). All experiments were performed in triplicate.

### NK Cell Functions

Effector functions of NK cells were assessed by flow cytometry. For target cell stimulation, 1 × 10^6^ PBMCs were mixed with K562 (ratio 10:1) for 4 h at 37°C and 5% CO_2_ as previously described (20). Unless otherwise specified, PBMCs were kept unstimulated before functional assays. When indicated, PBMCs were primed overnight with recombinant (r)IL-2 (50 UI/mL) and rIL-15 (5 ng/mL) prior to functional assays (Figure S2A in Supplementary Material). For cytokine production assays after cytokine stimulation, PBMCs were thawed, counted, and incubated for 18 h in the presence or absence of rIL-12 (5 ng/mL) and rIL-18 (20 ng/mL). Cells were then incubated with or without target cells with Golgiplug (BD Biosciences) for four additional hours, and then prepared for FACS analysis.

### Statistical Analysis

Statistical analyses were performed using GraphPad Prism software. For comparison between multiple matched samples, a Friedman test with a Dunn’s posttest was performed. For comparison between two independent groups, a Mann–Whitney test was performed. Statistical significance was indicated as *P < 0.05, **P < 0.01, and ***P < 0.001. In all graphs, data represent mean ± SEM.

## Results

### Reconstitution of Antitumor Effectors after CT

**Figure 1:**
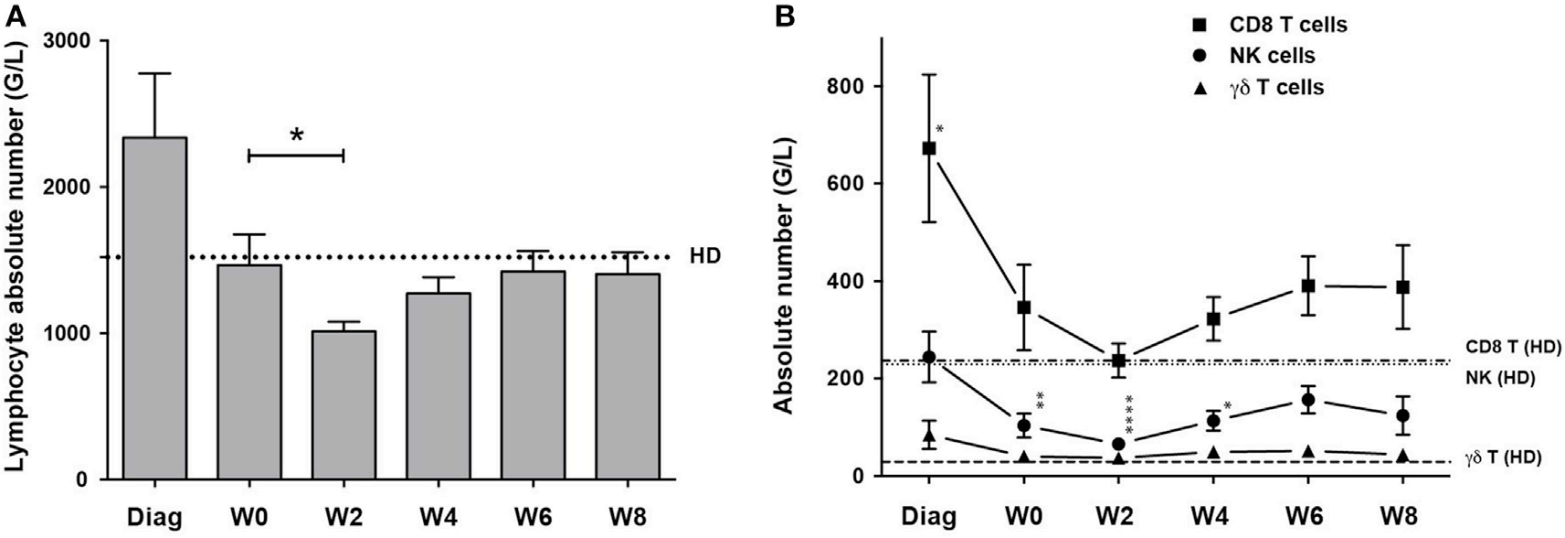
**Kinetics of cytotoxic lymphocyte reconstitution**. Kinetics of NK cells (round dots), CD8^+^ T cells (squares) and γδ T cells (triangles) reconstitution at the indicated time points. Cell counts (mean ± SEM) were measured at diagnosis of the disease, before the second consolidation (week 0, W0), at week 2, 4, 6, and 8 (W2, W4, W6, W8). **(A)** Absolute lymphocyte count. Dotted line corresponds to the mean of lymphocyte counts from 15 healthy donors (HD). **(B)** NK, γδ T, and CD8^+^ αβ T cell absolute counts. Horizontal lines correspond to the mean of NK, γδ T, and CD8^+^ αβ T cell counts from HD. When indicated a Kruskal–Wallis non-parametric test with Dunn’s posttest was performed to compute the *P* value for the comparisons (*P < 0.05, **P < 0.01, ****P < 0.0001).

We analyzed the peripheral blood of 29 patients diagnosed for a primary AML at diagnosis, after induction CT, and during second consolidation therapy cycle (Figure S1 in Supplementary Material and Section “Materials and Methods”). NK, γδ and CD8^+^ T cell absolute counts were calculated and compared to those of age-matched HDs. At diagnosis, lymphocyte counts were higher than that of HDs and were restored after CR (Figure 1A). As expected, 2 weeks after the second cycle of consolidation CT, lymphocyte counts significantly dropped but recovered by W6. CD8^+^ T cell numbers were higher at diagnosis, whereas NK and γδ cell numbers were comparable to control (Figure 1B). γδ T cell counts were back to normal after CR and remained constant. CD8 T cell counts dropped after induction and reached the levels of HD after the second consolidation, but rised again to stay high compared to HD (not significant). Similar to the other populations, NK cell counts dropped during induction and the first consolidation, eventually increased but cell counts never reached the levels of HD.

### Expression of Activating Receptors in Cytotoxic Effectors during CT

**Figure 2:**
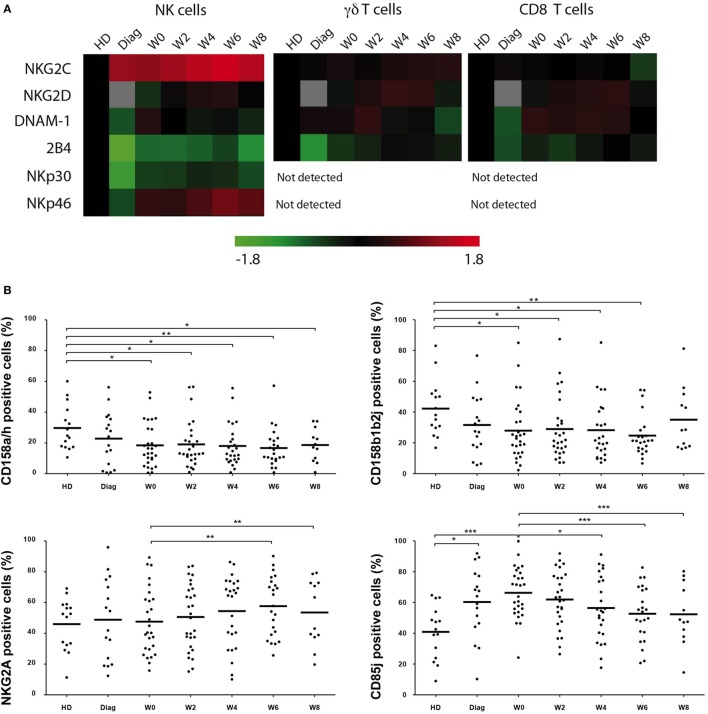
**Kinetics of activating and inhibitory receptor expression**. **(A)**. Global view of expression of activating receptors was measured by flow cytometry on NK, γδ T, and CD8^+^ αβ T cells at the indicated time points. MFI (unimodal expression) and percentage of positive cells (bimodal expression) were normalized and hierarchically clusterized with Tmev software. Comparison with healthy donors (HDs) was performed, and data are represented with colors reflecting higher (red) or lower (green) expression compared to HDs. Gray squares correspond to non-determined values. **(B)**. Expression of CD158a/h, CD158b1/b2/j, NKG2A, and CD85j by NK cells from HD and patients at the indicated time points. Bars represent the median expression and when indicated a Kruskal–Wallis non-parametric test with Dunn’s posttest was performed to compute the *P* value for the comparisons (*P < 0.05, **P < 0.01, ***P < 0.001).

We next analyzed whether expression of coactivating receptors was affected during the course of AML treatment (Figure 2A). NKG2C expression was found higher on NK cells from patients at diagnosis compared to HDs (*P* = 0.0029) and expression remained high during the treatment. DNAM-1 and 2B4 were found downregulated at diagnosis (*P* = 0.0024 and *P* < 0.0001, respectively). Interestingly 2B4 expression remained lower compared to HD during the study, whereas DNAM-1 expression was completely restored. NKG2D expression was unaffected during the study albeit the comparison with AML at diagnosis could not be tested. Finally, we confirmed in this study the downregulation of NKp30 and NKp46 at the diagnosis (*P* < 0.0001 and *P* = 0.0024, respectively). NKp30 expression was partially restored at W8 (MFI = 953 ± 134 vs HD: MFI = 1,304 ± 108, *P* = 0.0365), whereas that of NKp46 was completely restored, even higher than controls (W6: MFI = 3,340 ± 306 vs HD: MFI = 1,921 ± 212, *P* = 0.0017 and W8: MFI = 3,221 ± 465 vs HD: MFI = 1,921 ± 212, *P* = 0.0188). Noteworthy, neither CD56 nor CD16 expression was altered in patients at diagnosis or during treatment (data not shown). With respect to γδ T cells and CD8 T cells, NKG2C, NKG2D, DNAM-1, and 2B4 expression was also analyzed and revealed a completely different picture. 2B4 expression was downregulated at diagnosis (γδ T cells: MFI: 1,892 ± 237 vs HD: MFI = 2,300 ± 119, *P* = 0.0013; CD8 T cells: MFI: 1,640 ± 146 vs HD: MFI = 1,827 ± 123, *P* = 0.05) and restored at the end of the study. NKG2C, NKG2D, and DNAM-1 were expressed with similar frequencies and MFI compared to controls.

Overall, with respect to activating receptors, NK cells seemed to be the main effector population affected during the treatment of AML patients. We next decided to focus the study on NK cells.

### Expression of HLA Receptors in NK Cells during CT

We next analyzed the expression (frequency) of HLA receptors, i.e., NKG2A, CD85j, and KIR/CD158, on NK cells from patients under treatment (Figure 2B). The anti-CD158 antibodies used here do not discriminate between inhibitory and activating KIRs. Our analyses revealed a downregulation of CD158a/h and CD158b1/b2/j at diagnosis and after reaching CR (W0) compared to HD. NKG2A expression was not drastically affected during time compared to controls at all time points, although there was some increase at W6 and W8 compared to CR. In contrast, CD85j was more frequent on NK cells from patients at diagnosis and before the second cycle of consolidation CT. Expression tended to decrease over time during treatment, although remaining slightly higher than HD. Of note, frequencies of HLA receptors in γδ T cells and CD8 T cells were slightly altered during the study, with a non-significant increase of CD158b1/b2/j and CD85j (Figure S2 in Supplementary Material).

### Kinetics of Early Stages of NK Cell Maturation during CT

**Figure 3:**
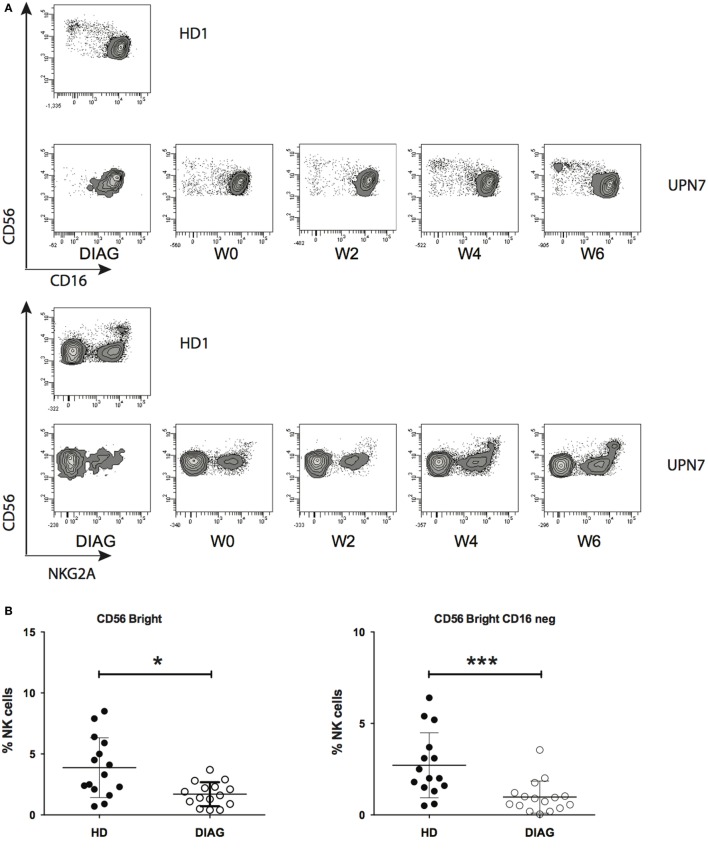

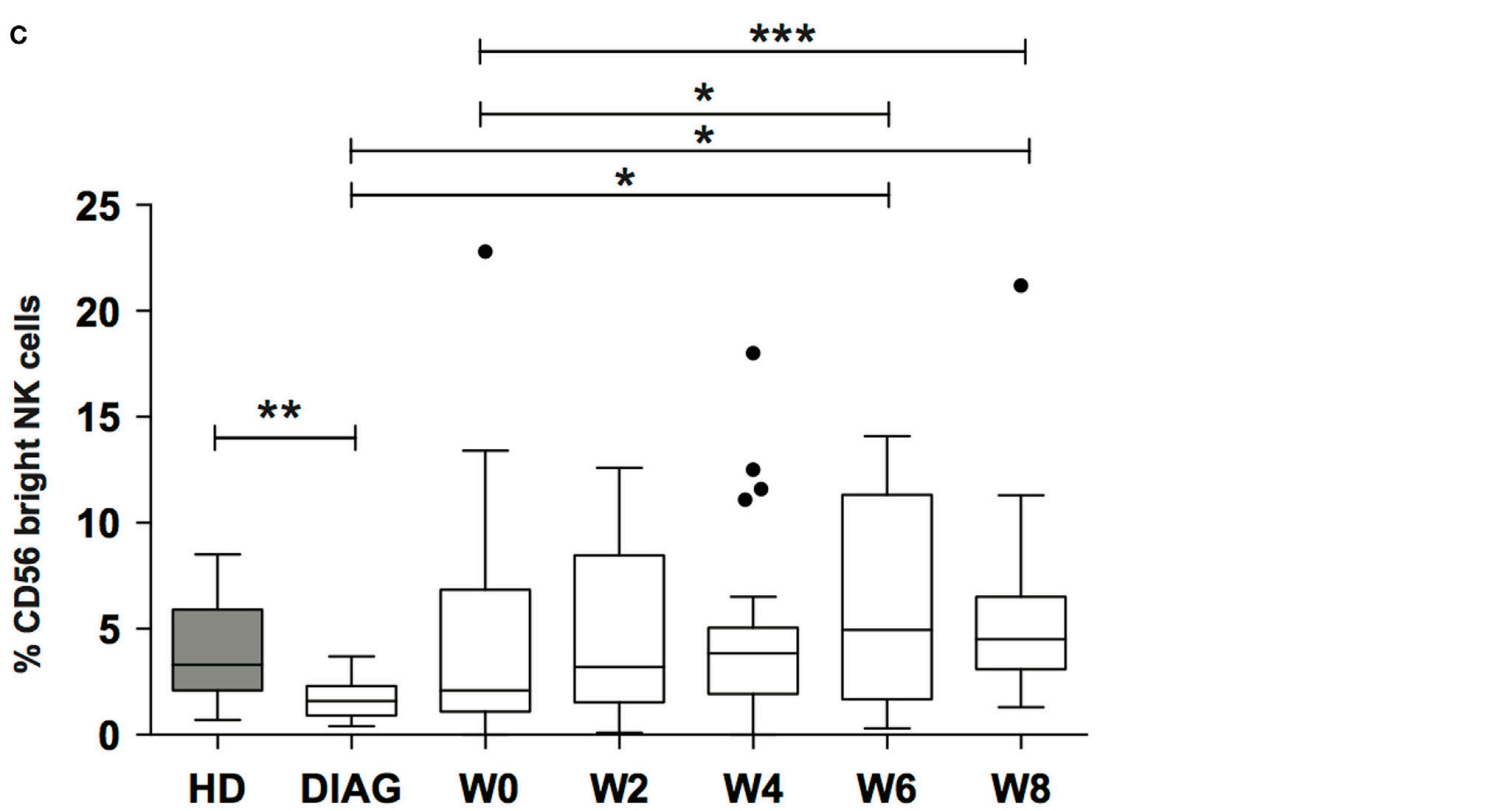
**Kinetics of immature NK cells reconstitution**. **(A)** Example of reconstitution of CD56^bright^ NK cells in a patient following treatment. The figure displays the reapparition of CD56^bright^ NK cells expressing or not CD16 (top) or NKG2A (bottom). **(B)** Frequencies of total CD56^bright^ NK cells (left panel) or CD56^bright^ NK cells lacking CD16 expression (right panel). **(C)** Summary figure of reconstitution of the immature CD56^bright^ NK cell subset after induction and consolidation therapies. Data represent Tukey whisker boxes, with outliers represented as round dots. When indicated a Kruskal–Wallis non-parametric test with Dunn’s posttest was performed to compute the *P* value for the comparisons (*P < 0.05, **P < 0.01, ***P < 0.001).

We next sought to analyze whether NK cell early maturation was normal in patients before and after AML treatment. Combinations of CD56, CD16, and NKG2A define different steps of NK cell maturation (4, 5). CD56 expression as well as frequencies of CD16 and NKG2A positive cells out of total NK cells were comparable to HD (data not shown and Figure 2B). However, it is known that NKG2A is expressed by all CD56^bright^ NK cells and CD16 expression defines two subsets (4). CD56^bright^ NK cells are usually a rare population compared to CD56^dim^ NK cells, and therefore, information about these cells may be masked when looking at the total NK level. Therefore, we analyzed the combinations of these three markers in order to identify the different maturation subsets based on CD56, CD16, and NKG2A markers. We observed a profound loss of total CD56^bright^ and CD56^bright^ CD16^−^ NK cells at diagnosis [Figures 3A,B (left and right panels, respectively); Figure S3 in Supplementary Material]. After induction and reaching CR, total CD56^bright^ were present at low frequency and continued to rise during consolidation (Figure 3C).

### Functional Properties of NK Cells after CT

**Figure 4:**
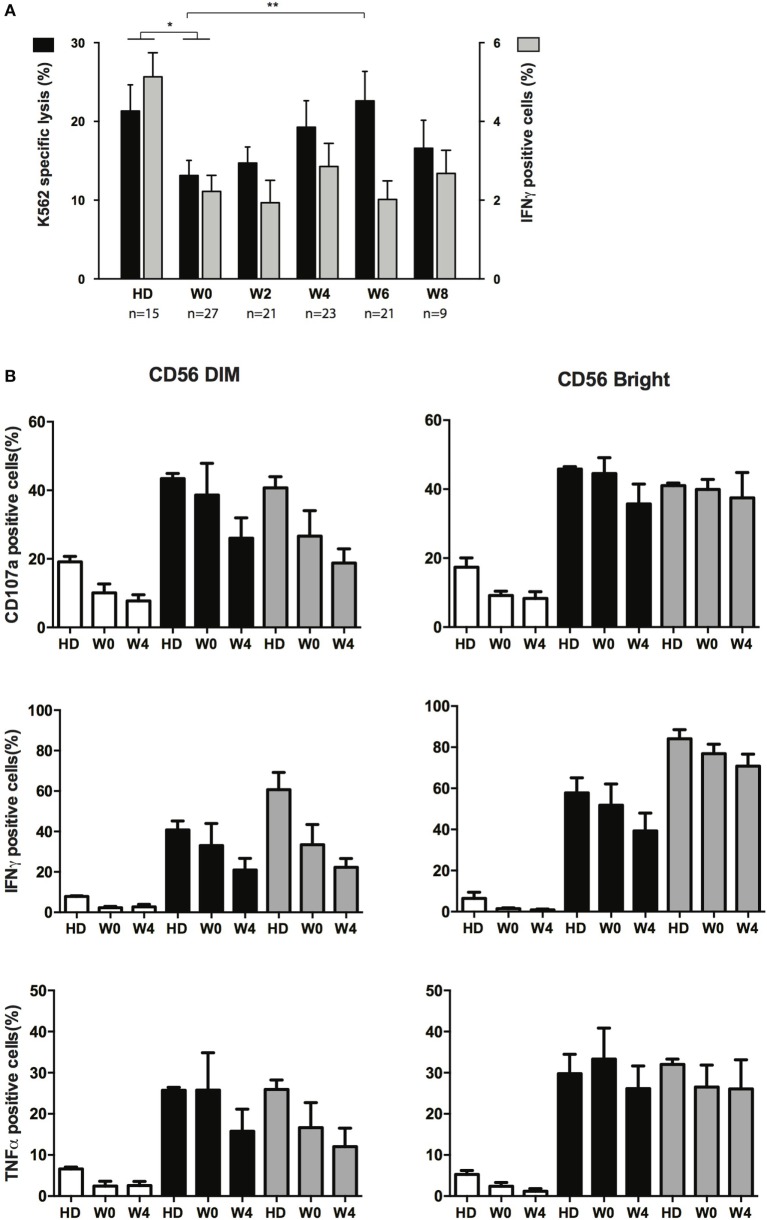
**Effector functions of NK cells following chemotherapy**. **(A)** Specific lysis of target cells (black bars, left axis) and intracellular production of IFN-γ (gray bars, right axis) after interaction between NK from healthy donors (HDs) or patients at the indicated time points and K562 cells. Histogram represents mean and bars represent SEM. **(B)** Effect of cytokine priming on degranulation and production of IFN-γ and TNF-α upon interaction with K562 cells. NK cells from HDs (*n* = 3) and patients (*n* = 5) at the indicated time points were treated overnight with medium only (white bars), IL-2 + IL-15 (black bars) or IL-12 + IL-18 (gray bars). Cells were then washed and incubated for 4 h with K562 cells. Effector responses were analyzed as indicated in the Section “Materials and Methods.” The figure depicts CD56^dim^ (left) and CD56^bright^ (right) identified with the flow cytometry analysis software.

NK cells from AML patients have defective cytolytic activities (11, 14, 21). We next sought to verify whether cytotoxicity was restored during treatment and whether other functions of NK cells were affected during treatment by CT. Degranulation (CD107a) and IFN-γ or TNF-α production assays were performed after interaction with K562 cells. Degranulation capacities of total NK cells during recovery could not be compared to NK cells at diagnosis, due to the paucity of NK cells, and was thus compared to HD or total NK cells at CR. Degranulation was impaired in NK cells from patients in CR (W0) but was, at least partially, restored during consolidation (Figure 4A, black bars). Degranulation never reached the level of HD, and at W8, NK cells displayed a somewhat reduced degranulation capacity compared to W6 (non-significant). Interestingly, despite reduced degranulation at early time points, we found that perforin content in NK cells (and T cells) was higher compared to HD (Figure S4 in Supplementary Material). Similar to degranulation, IFN-γ production was altered in patients in CR, but we did not observe any restoration of the capacity of NK cells to produce IFN-γ (Figure 4A, gray bars) and TNF-α (not shown) after interaction with K562 compared to HD. In order to verify whether the whole cytokine production machinery was impaired, and not only the capacity to respond to target cells, we incubated overnight NK cells with a cocktail of IL-2/IL-15 or IL-12/IL-18 and then incubated these NK cells with K562 cells (Figure 4B). As CD56^bright^ NK cells and CD56^dim^ NK cells were identifiable at W0 (in contrast to diagnosis), these two subsets were gated and analyzed. After interaction with target cells, both CD56^dim^ and CD56^bright^ NK cells from patients at W0 pretreated with IL-2/IL-15 or IL-12/IL-18 displayed a significant increase in degranulation as well as IFN-γ and TNF-α production, similar to controls. Interestingly, functions of cytokine-primed CD56^dim^ NK cells at W4 (4 weeks after the second consolidation cycle) were somewhat decreased compared to functions measured at W0. Regarding CD56^bright^ NK cells, a slight decrease of CD107a and IFN-γ was observed, but generally, the effect was minor compared to CD56^dim^ NK cells.

**Figure 5:**
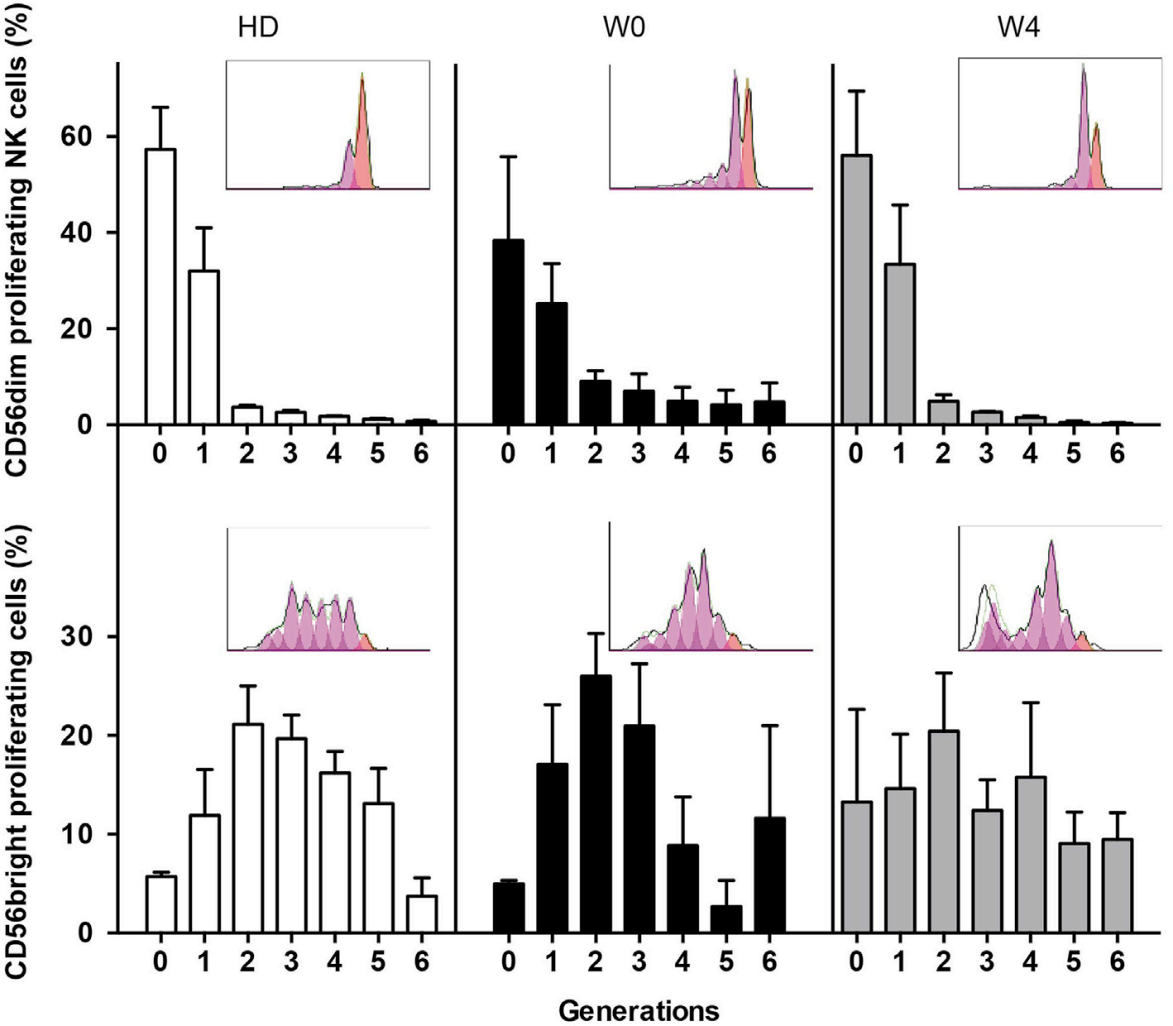
**Proliferation capacities of NK cells before and during consolidation**. Proliferation capacities of NK cells during chemotherapy (CT). NK cells from healthy donors or patients before (W0) and after the first cycle of consolidation CT were isolated from peripheral blood, stained with CellTrace Violet, and cultured for 6 days in IL-2-containing medium. The figure displays CellTrace Violet dilution reflecting cell proliferation for CD56^dim^ NK cells (top) and CD56^bright^ NK cells (bottom). The histograms indicate the frequency of non-proliferating cells (generation 0) and proliferating cells (generations 1–6). Data represent mean ± SEM. Insets show a representative donor or patient (*n* = 3 and *n* = 5, respectively).

Finally, we analyzed the capacity of NK cells to proliferate in response to IL-2 and IL-15 stimulation (Figure 5). By means of CellTrace Violet dilution assay, we observed that NK cells from patients at W0 and W4 had a high capacity of proliferation, similar to controls. As expected, CD56^bright^ NK cells proliferated more than CD56^dim^ NK cells as revealed by the high frequency of cells with longer history of cell division (>2 generations). Of note, NK cells from patients at diagnosis were not analyzed because of the lack of CD56^bright^ NK cells. Altogether these data revealed that NK cell effector functions that can be triggered by target cells are altered at early steps of AML treatment and that only degranulation is restored over time. By contrast, the sensitivity to exogenous cytokine stimulation was not affected by consolidation CT.

## Discussion

The treatment of hematological malignancies, including AML, requires the standardized and recommended use of anthracyclines and aracytine (17). Frequent relapse is the proof that the initial treatment is not sufficient and leaves the patient with a minimal residual disease that eventually overcomes immune surveillance, leading to relapse. Little is known about the status of the immune system during the early steps of CT in AML, in particular with NK cells. Here, we studied effector lymphocytes (NK, CD8, γδ T cells) frequencies during the early course of treatment of elderly AML patients. We analyzed the expression of the main activating and inhibitory receptors in these cells. Focusing on NK cells, we have analyzed their effector functions in response to target cell or cytokine stimulation. Our data revealed that NK cells are the main population affected at diagnosis or during treatment. Although NK cells expand after the induction CT, they remain probably more sensitive to the consolidation CT because the NK cell counts remained lower than that of HD, as described before (22). In contrast, CD8 T cells and γδ T cells were more frequent than in HDs during the entire study. In addition, NKG2C, DNAM-1, 2B4, and NKG2D were expressed on all T cells at similar levels compared to HD. In contrast, confirming previous studies, DNAM-1, 2B4, NKp30, and NKp46 were downmodulated at diagnosis on NK cells. The impact of treatment resulted in a partial or total restoration of NKp30 and NKp46, respectively, as previously shown (14), and a restoration of DNAM-1 expression. Surprisingly, 2B4 expression remained low during the 8 weeks of observation. The fact that the alterations mostly concerned NK cells likely reflects their involvement in the control of leukemia progression. Alternatively, the general downmodulation of NK-activating receptors shed light on the suppressive pathway that leukemia use to hamper innate immune recognition. Several mechanisms have been suggested explaining how cancer cells impair NK cell functions, such as TGFβ-1 production by tumor cells (10), or the production of histamine and reactive oxygen species by phagocytes (23). A phase III clinical trial using histamine dihydrochloride in addition to IL-2 improved the leukemia-free survival of AML patients (24). Alternatively, it was suggested that AML-induced alteration of NK cells was mediated by IL-10 (21). Our study, along with previous studies, suggests that chemoresistant remaining tumor cells may remain untargeted by defective NK cells. Hence, the weak expression of activating NK receptors is expected to result in a lack of recognition of residual tumor cells. An interesting parameter to analyze would be to compare the level of NK cell receptor expression with the incidence of relapse. In line, a recent studies identified correlations between NK cell receptor profiles with OS and relapse risk (25).

In accordance with defective receptor expression at diagnosis, degranulation, and IFN-γ production upon interaction with target cells were defective at diagnosis. Beside intrinsic insufficiency to degranulate against classical HLA-negative NK target cells, AML-NK cells, and also AML-T cells, display a reduced capacity to form solid conjugates and effective immune synapse with leukemic cells (26, 27). Albeit degranulation properties were restored over time, cytokine production remained impaired along the time course of the study. These data suggest that NK cell recovery after CT is incomplete. Interestingly, at W4 functions of NK cells after exogenous cytokine and target cell stimulation was further decreased compared to W0. Although this finding should be confirmed, it suggests that at this time unknown factors may delay the reconstitution of NK cell function. These data are similar to the reconstitution of NK functions after stem cell transplantation (SCT) where degranulation is functional but not pro-inflammatory cytokine production (1, 28). It seems that degranulation capacities of NK cells during immune reconstitution are a faster process compared to regulatory functions. These data warrant more investigation such as longer time points of observation since in SCT it takes up to 1 year to fully recover IFN-γ or TNF-α production capabilities (1). Furthermore, it would be interesting to determine whether, like for SCT, a fast cytokine production recovery correlates with a lower probability of relapse.

In addition to activating NK receptors, we also analyzed the expression of HLA receptors. Expression of classical HLA-I receptors (i.e., KIRs) was downmodulated in NK cells at the diagnosis and CR, but was restored over time, suggesting a potential break of tolerance, which is unfortunately hampered by the downregulation of activating receptors. NKG2A was not affected at diagnosis nor during treatment. Thus, NK cell activation remains tightly controlled, likely by tumor cells, keeping NK cells tolerant to remaining tumor cells.

Despite the normal expression of CD16 and NKG2A, we could not exclude alterations in the development of NK cells both at diagnosis and during the treatment, since CT induces aplasia. For instance, after HSCT, the population of immature NK cells is prominent during several months after graft (1, 29). Surprisingly, when we measured the ratio between CD56^bright^ NK cells (immature) and CD56^dim^ NK cells, we observed a dramatic reduction in the frequencies of CD56^bright^ NK cells, and particularly in the most immature CD56^bright^ CD16^−^ NK cells. The fact that these cells, as well as CD56^bright^ CD16^+^ NK cells, recover over time suggest that leukemic cells have somehow a deleterious effect on this population. In our cohort, we observed an increase of CD56^bright^ NK cell frequency between the diagnosis and W0 just before the second induction and a continued increase until the end of study. This observation suggests that consolidation CT has little impact on NK cell reconstitution. We cannot exclude that abnormal NK cell differentiation is maintained at these early time point from induction as the cohort is too small. Our observation seems to contradict the study by Dauguet et al. showing that most patients at first CR displayed an unusual high frequency of CD56^bright^ NK cells (30). However, we did not analyze our patients during this very early phase of treatment (15–30 days post induction CT), and we could have missed a critical period of time where NK cells undergo rapid expansion and differentiation as a sign of homeostatic proliferation. This subversion of NK cell maturation by tumor cells has been observed in other cancer settings such as breast cancer (31). Interestingly, a study by Harlin et al. suggested that the microenvironment may alter NK cell biology and notably survival by producing reactive oxygen species, which kill CD56^dim^ NK cells but somehow spare CD56^bright^ NK cells (32). It would be interesting to test whether metabolism, particularly oxygen metabolism, is altered during these early phases of AML treatment. Obviously other mechanisms may be involved. In line, studies in mouse models have also evidenced a direct impact of tumor cells, including AML-like cancer, on NK cell maturation (33, 34). Importantly, the recent study by Mundy-Bosse et al. also showed that CD56^bright^ NK cells are less frequent in AML patients at diagnosis (34). Thus, our study confirms the study of Mundy-Bosse et al. Interestingly, the microRNA mir-29b seems to be involved in the process of maturation blockade both in mice and humans (34). EOMES and T-BET are targets of Mir-29b, and these transcription factors are critical for early NK cell differentiation. Nonetheless, studies are required to elucidate the link between leukemic cells and mir-29b or other potential targets of leukemic cells.

A more thorough analysis with additional differentiation markers, such as combination of KIRs, NKG2A, and CD57, should be performed in order to identify the extent of NK cell maturation recovery during consolidation therapy. Addressing the mechanisms of such a defect would be difficult in humans. A blockade of NK cell differentiation would likely result in a defective production of mature NK cells, which is not the case in our cohort and according to previous studies. Oppositely, an acceleration of maturation of NK cells, while preserving a normal production of NK cells, would potentially result in an accumulation of more mature NK cells. Accordingly, in a different cohort, we have observed an accumulation of late-stage matured CD56^dim^ NK cells expressing the markers CD57 and KIRs in AML patients at diagnosis (35). Interestingly, these two populations (i.e., immature CD56^bright^ and the most mature CD56^dim^ KIR^+^ CD57^+^ NK cells) are distinguished by their differential capacities to respond to cytokines and to regulate other immune cells *via* cytokine production capacities (5, 36). The reduction of CD56^bright^ NK cell pool could then participate to the immune tolerance to leukemic cells. Interestingly, this dichotomy between CD56^bright^ and CD56^dim^ ratio has been recently associated with additional defects in NK cells and correlates with clinical outcome of patients and may have potential consequences on the results of future NK cell-based immunotherapies (25). It seems of importance to note that at W0, CD56^bright^ NK cells that had reappeared behaved in a comparable way to HDs’ CD56^bright^ NK cells. Hence, cytokine production, degranulation, and proliferation capacities were similar. These data suggest that these cells may have recovered fully, or simply that the blockade of maturation is paralleled but not linked to alterations of phenotype and functions.

Our data show that NK cells are present in almost normal numbers in patients with AML in CR and NK cells remain present after consolidation CT. Our data provide new insights in the optimal period to introduce NK cell-based immunotherapy, i.e., when NK cells have recovered sufficient effector functions such as cytokine production and cytotoxicity. Many NK cell-based immunotherapies have been developed over the last decades, with allogeneic or autologous NK cells (37). In 2005, haploidentical NK cells were administered in a non-transplantation setting and resulted in a substantial improvement of patient clinical outcome (38). More recently, *in vivo* targeting of NK cells with antibodies was investigated: IPH2101 is a first-in-class anti-KIR mAb that blocks inhibitory KIR–ligand interactions, leading to restoration of NK cell functions (39). A phase II trial in AML elderly patients in first CR1 (NCT01687387) is in progress and several other trials are ongoing in different cancers alone or in combination with other treatments. The future introduction of a first-in-class anti-NKG2A blocking antibody (IPH2201) will also provide a novel strategy to enhance tumor cell recognition (40). Additionally, recognition of leukemic cells by NK cells may be improved by the use of newly engineered antibodies such as CD16xCD33 bispecific antibodies (41). Finally, IMIDs such as lenalidomide represent another immunotherapy enhancing NK and T cell recognition of leukemic cells (26, 42).

Altogether, we present the first longitudinal study allowing determining which time window may be optimal to proceed to the most up-to-date NK cell-based immunotherapies for elderly patients excluded from conventional allogeneic SCT.

## Ethics Statement

All subjects gave written informed consent in accordance with the Declaration of Helsinki.

## Author Contributions

JR designed the study, performed experiments, analyzed the data, and wrote the manuscript; CF analyzed the data, performed statistical analyses, and wrote the manuscript; EK and FO performed experiments; BB analyzed the data and performed statistical analyses; AC and ED provided samples and clinical expertise; NV, PA, and FR designed the study and wrote the manuscript; NV and DO designed and supervised the study and wrote the manuscript.

## Conflict of Interest Statement

PA is an Innate Pharma employee. FR is the former CSO of Innate Pharma and a former employee of Innate Pharma. The remaining authors declare no conflict of interest.

## Supplementary Material

Figure S1 **Timeline of the study**. We enrolled 29 patients from diagnosis of acute myeloid leukemia (AML) and followed them during the treatment of AML. Peripheral blood samples were taken at diagnosis, in complete remission, before the second consolidation CT (W0) and every other week (W2, W4, W6, W8) after treatment for 8 weeks.

Figure S2 **Expression of inhibitory receptors by αβ and γδ T cells**. Expression of CD158a/h, CD158b1/b2/j, NKG2A, and CD85j by αβ and γδ T cells from healthy donors (HDs) and patients at the indicated time points. Bars represent the median expression of the indicated markers. Horizontal lines correspond to the mean expression of HDs (*n* = 15).

Figure S3 **Expression of inhibitory receptors by αβ and γδ T cells**. Gating strategy of NK cells from patients at diagnosis and during treatment. One representative patient is shown at diagnosis and week 6.

Figure S4 **Expression of perforin by NK cells and αβ and γδ T cells**. Expression of perforin was measured by intracellular staining and flow cytometry analysis on the indicated populations from peripheral blood of patients at the indicated time points. Histograms represent mean ± SEM. Horizontal lines correspond to the mean expression of perforin in cells from healthy donors (*n* = 15).

## Abbreviations

NK, natural killer; AML, acute myeloid leukemia; CR, complete remission; CT, chemotherapy; HDs, healthy donors; KIRs, killer cell immunoglobulin-like receptors.
