# Supplementary figures and images for: Kinetics of Cytotoxic Lymphocytes Reconstitution after Induction Chemotherapy in Elderly AML Patients Reveals Progressive Recovery of Normal Phenotypic and Functional Features in NK Cells
Source: Front Immunol. 2017 Feb 2;8:64. doi: 10.3389/fimmu.2017.00064 (PMC5288405; doi:10.3389/fimmu.2017.00064)

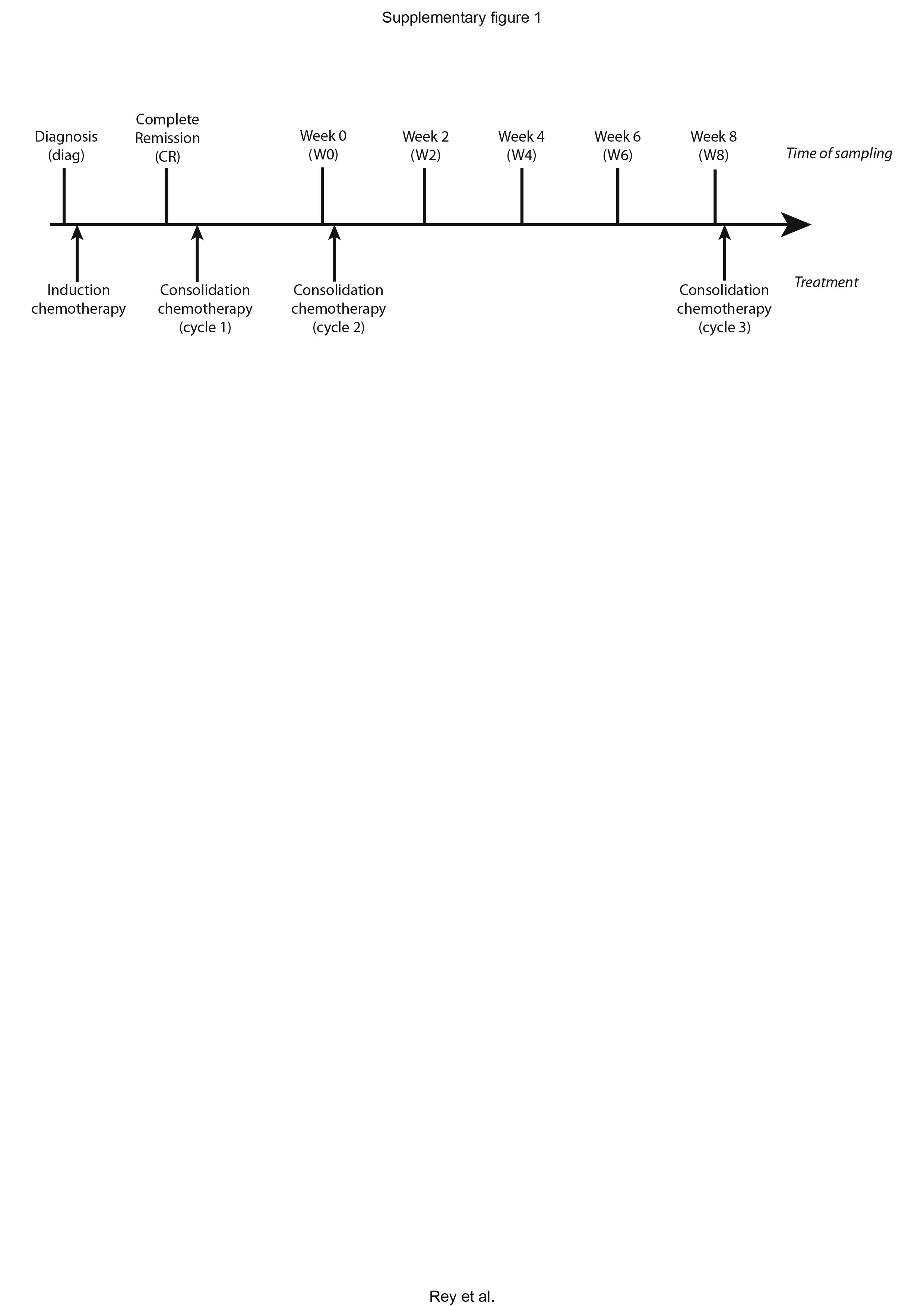

Supplement: Figure S1 — Timeline of the study. We enrolled 29 patients from diagnosis of acute myeloid leukemia (AML) and followed them during the treatment of AML. Peripheral blood samples were taken at diagnosis, in complete remission, before the second consolidation CT (W0) and every other week (W2, W4, W6, W8) after treatment for 8 weeks. [file image_1.jpeg]

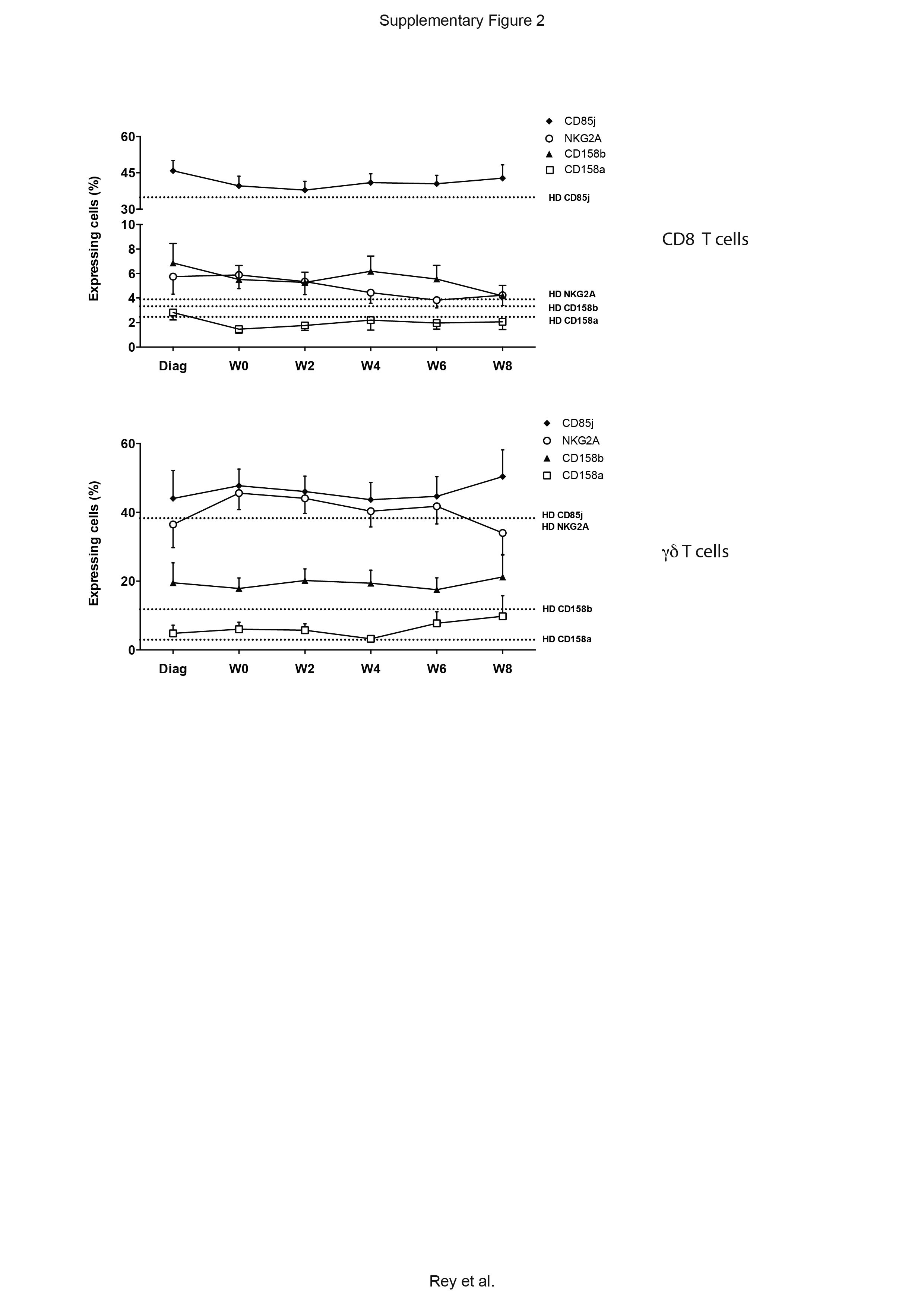

Supplement: Figure S2 — Expression of inhibitory receptors by αβ and γδ T cells. Expression of CD158a/h, CD158b1/b2/j, NKG2A, and CD85j by αβ and γδ T cells from healthy donors (HDs) and patients at the indicated time points. Bars represent the median expression of the indicated markers. Horizontal lines correspond to the mean expression of HDs (n = 15). [file image_2.jpeg]

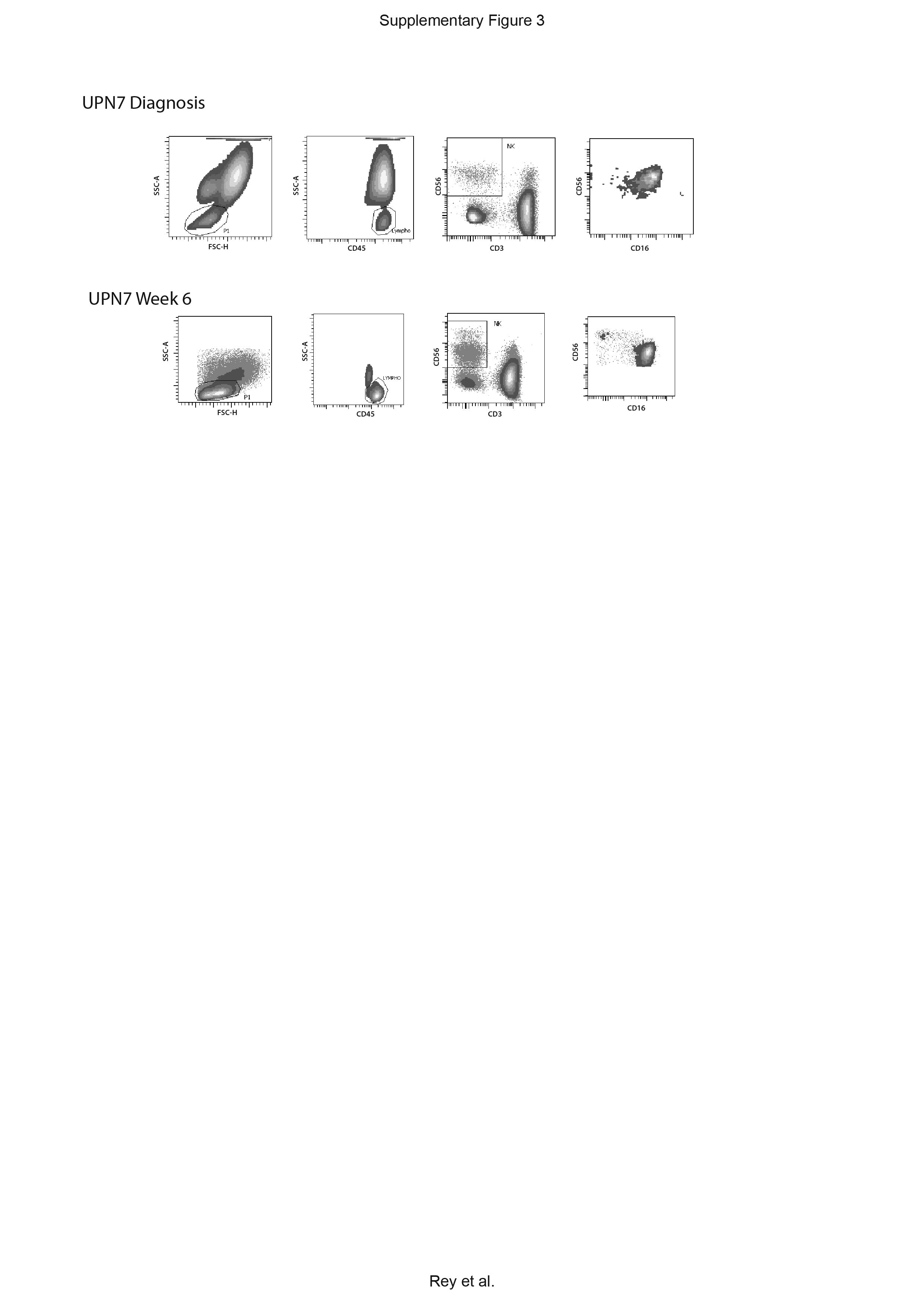

Supplement: Figure S3 — Expression of inhibitory receptors by αβ and γδ T cells. Gating strategy of NK cells from patients at diagnosis and during treatment. One representative patient is shown at diagnosis and week 6. [file image_3.jpeg]

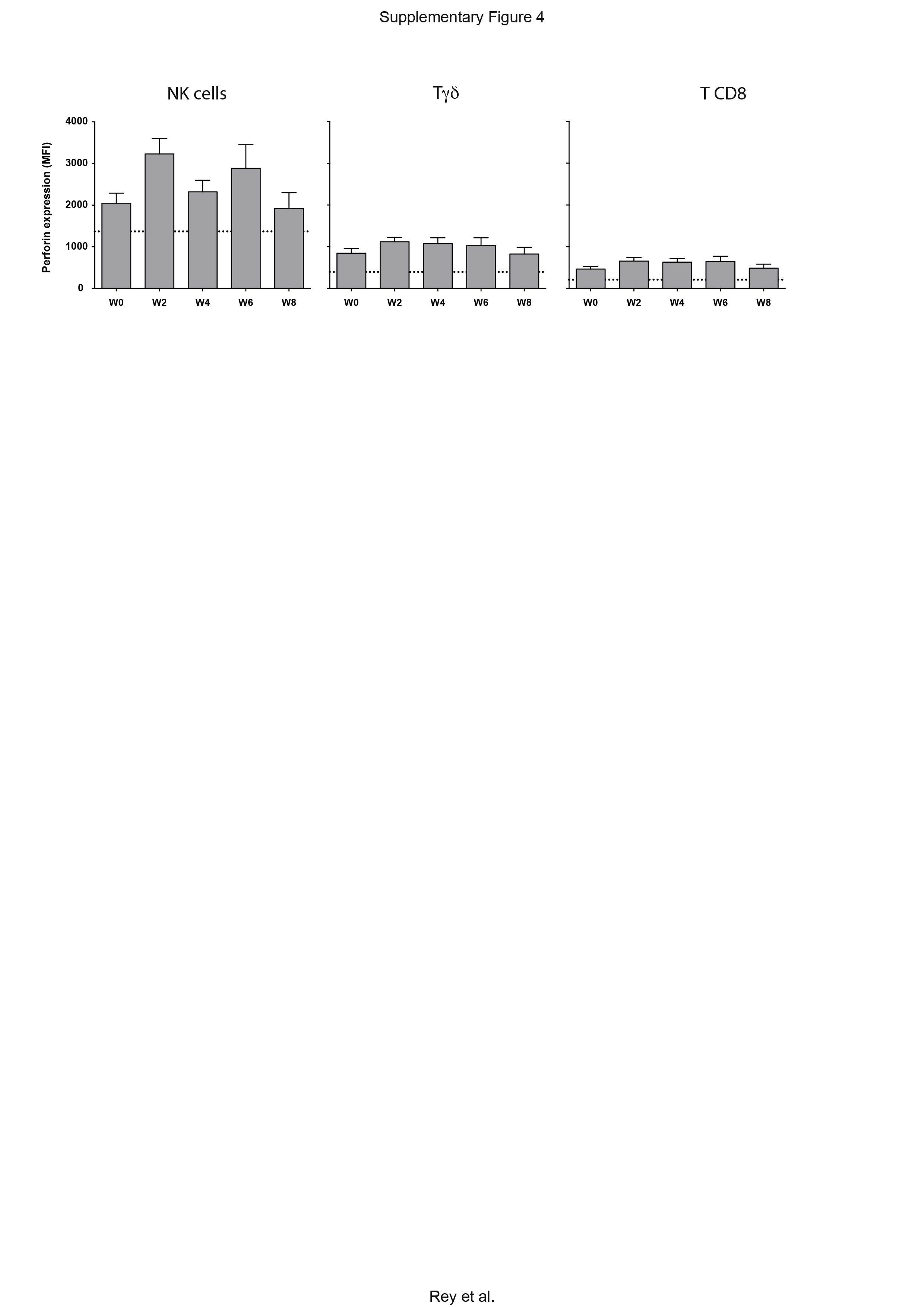

Supplement: Figure S4 — Expression of perforin by NK cells and αβ and γδ T cells. Expression of perforin was measured by intracellular staining and flow cytometry analysis on the indicated populations from peripheral blood of patients at the indicated time points. Histograms represent mean ± SEM. Horizontal lines correspond to the mean expression of perforin in cells from healthy donors (n = 15). [file image_4.jpeg]
