# Supplementary material for: Kinetics of Cytotoxic Lymphocytes Reconstitution after Induction Chemotherapy in Elderly AML Patients Reveals Progressive Recovery of Normal Phenotypic and Functional Features in NK Cells
Source: Front Immunol. 2017 Feb 2;8:64. doi: 10.3389/fimmu.2017.00064 (PMC5288405; doi:10.3389/fimmu.2017.00064)
Supplement: Supplementary file 5 [file table_1.doc]

**Supplementary Table 1.** Antibodies and reagent used for Flow cytometry used in the study. All flow cytometry experiments were acquired on a FACS Canto II (Becton-Dickinson).

| **Antibodies/Reagents** | **Fluorochrome** | **Supplier** |
| --- | --- | --- |
| CD3 | Pacific Blue | Becton-Dickinson |
| CD3 | APC | Beckman-Coulter |
| CD56 | PE-Cy7 | Becton-Dickinson |
| CD19 | PE-Cy5 | Becton-Dickinson |
| CD14 | PE-Cy5 | Becton-Dickinson |
| CD16 | APC-H7 | Becton-Dickinson |
| CD158a,b,e,i | PE | Beckman-Coulter |
| CD85j | PE | Beckman-Coulter |
| NKG2A | PE | Beckman-Coulter |
| NKp30 | PE | Beckman-Coulter |
| NKp46 | PE | Beckman-Coulter |
| NKp44 | PE | Beckman-Coulter |
| NKG2D | PE | Beckman-Coulter |
| 2B4 | PE | Beckman-Coulter |
| DNAM-1 | PE | Beckman-Coulter |
| NKG2C | PE | Beckman-Coulter |
| Perforin | PE | Beckman-Coulter |
| Granzyme B | PE | Beckman-Coulter |
| 7-AAD | - | Becton-Dickinson |
| Cell Trace Violet | - | Life technologies |
| CD107a/b-FITC | FITC | Becton-Dickinson |
| IFN-γ | PE | Becton-Dickinson |
| TNF-α | APC | Becton-Dickinson |
